# Autoantibodies against type I interferons in patients with zoonotic H7N9 influenza: an observational case–control study

**DOI:** 10.1016/j.ebiom.2026.106387

**Published:** 2026-07-16

**Authors:** Yongkun Chen, Jakob Ankerhold, Tingting Jia, Martin Wolkewitz, Jonas Fuchs, Lifang Yuan, Tian Bai, Kevin Groen, Roger Kuratli, Benjamin G. Hale, Georg Kochs, Dayan Wang, Martin Schwemmle, Yuelong Shu, Laura Graf

**Affiliations:** a Guangdong Provincial Key Laboratory of Infection Immunity and Inflammation, School of Basic Medical Sciences, Shenzhen University Medical School, Shenzhen University, 1066 Xueyuan Avenue, Nanshan District, Shenzhen, 518055, China; b Institute of Virology, Medical Center - University of Freiburg, Hermann-Herder-Str. 11, Freiburg, 79104, Germany; c Faculty of Medicine, University of Freiburg, Freiburg, Germany; d University of Freiburg Spemann Graduate School of Biology and Medicine, Freiburg, Germany; e School of Public Health, Shenzhen Campus of Sun Yat-sen University, No. 66, Gongchang Road, Guangming District, Shenzhen, 518107, China; f Shenzhen Center for Disease Control and Prevention, No. 8, Longyuan Road, Nanshan District, Shenzhen, 518055, China; g Institute of Medical Biometry and Statistics, Medical Center - University of Freiburg, Freiburg, Germany; h Key Laboratory of Pathogen Infection Prevention and Control (MOE), State Key Laboratory of Respiratory Health and Multimorbidity, National Institute of Pathogen Biology, Chinese Academy of Medical Sciences & Peking Union Medical College, No. 16 Tianrong Street, Daxing District, Beijing, 102629, China; i Institute of Medical Virology, University of Zurich, Winterthurerstrasse 190, Zurich, 8057, Switzerland; j Chinese National Influenza Center, National Institute for Viral Disease Control and Prevention, Chinese Center for Disease Control and Prevention, 155 Changbai Road, Changping District, Beijing, 102206, China

**Keywords:** Type I interferon, Autoantibodies, Influenza A virus, Zoonosis, H7N9

## Abstract

**Background:**

The determinants of the species barrier preventing human infections with avian influenza A viruses (IAV) are incompletely understood. We previously identified loss-of-function variants of the interferon-regulated antiviral factor MxA as a genetic factor for increased susceptibility to infections with the H7N9 subtype. Given the central role of type I IFNs (IFN-I) in antiviral defence, we hypothesised that IFN-I-neutralising autoantibodies may similarly predispose to zoonotic H7N9 infection.

**Methods:**

In this observational case–control study, serum samples collected between 2013 and 2017 from 199 Chinese patients with laboratory-confirmed H7N9 infection and 531 healthy, uninfected controls (269 poultry workers, 262 close contacts) were screened for IgG autoantibodies binding IFNα_2_, IFNβ_1b_, or IFNω using a multiplex bead-based assay. Positive samples were tested for IFN-neutralising activity in a luciferase-based reporter assay. To confirm their ability to block IFNα_2_-mediated antiviral activity, selected samples (n = 19) were analysed in IAV infection experiments. Associations between age, sex, H7N9 case status, case fatality, and the presence of neutralising autoantibodies were evaluated by logistic regression. Available whole-genome sequencing data from 26 individuals with neutralising autoantibodies were screened for variants in genes linked to IFN-I autoimmunity.

**Findings:**

Neutralising autoantibodies against at least one IFN-I were detected in 19.1% (38/199) of patients but in only 1.1% (6/531) of controls, consistent with published general population data. Most patient sera targeted IFNα_2_ and/or IFNω (35/199), and 18.1% (36/199) neutralised even high IFN-I concentrations of 1–10 ng/ml. The presence of neutralising autoantibodies was associated with 8.2- to 25.3-fold higher odds of H7N9 infection (p < 0.0001), depending on antibody specificity and reference group. Autoantibody prevalence increased significantly with age in patients (44.8% ≥70 years; OR = 1.05; 95% CI 1.02–1.07; p = 0.0001), but was not associated with sex (OR for males vs. females = 0.52; 95% CI 0.23–1.14; p = 0.106). All selected sera containing neutralising autoantibodies blocked IFNα_2_-induced antiviral activity in cell culture. No known genetic predisposition for IFN-I autoimmunity was identified.

**Interpretation:**

Our findings suggest that IFN-I-targeting autoimmunity is associated with susceptibility to zoonotic IAV infection with the H7N9 subtype, and possibly also other subtypes, including panzootic H5N1. Given the ease of implementation, screening for anti-IFN-I autoantibodies could be readily integrated into surveillance or targeted testing. This could be relevant in environments with increased exposure to zoonotic IAVs.

**Funding:**

Shenzhen Medical Research Fund, National Natural Science Foundation of China, Non-profit Central Research Institute Fund of Chinese Academy of Medical Sciences, Guangdong Provincial Science and Technology Program, Program for Youzuzhikeyan of Shenzhen University, German Research Foundation, Swiss National Science Foundation.

**Research in context**

**Evidence before this study**

The type I interferon (IFN-I) system is a key component of the antiviral defence against influenza A viruses (IAVs). Genetic studies have identified rare inborn errors of IFN-I immunity that increase susceptibility to zoonotic H7N9 infection demonstrating its importance in the avian-human species barrier. A PubMed search including the search terms “avian”, “influenza A virus”, “autoantibodies”, and “interferon” for articles published before Aug 15, 2023 (with updates in Aug 2024, Aug 2025, and March 2026) found no prior studies assessing whether anti-IFN-I autoantibodies contribute to human susceptibility to avian IAV infections. Available literature at that time was limited to reports showing that IFN-I-neutralising autoantibodies predispose individuals to severe disease after infection with seasonal IAVs and other viruses. A recently published case report (Dec 2025) describing IFN-I-neutralising autoantibodies in a single patient who died from H5N1 avian influenza further indicates that IFN-I autoimmunity may play a role in avian IAV spillover events.

**Added value of this study**

This study provides evidence that IFN-I-neutralising autoantibodies are strongly enriched in individuals infected with an avian IAV and are able to impair the IFN-I-mediated antiviral response. We show that the presence of these autoantibodies is associated with increased susceptibility to H7N9 infection. These findings extend the role of IFN-I autoimmunity from influencing viral disease severity to shaping susceptibility to zoonotic infection, further supporting the central role of the IFN-I system in the avian-human species barrier.

**Implications of all the available evidence**

Our findings indicate that exposed individuals with pre-existing IFN-I-neutralising autoantibodies show increased vulnerability to zoonotic, avian IAV infections. The prevalence of such autoantibodies is considerably higher in the general population than previously described rare genetic risk variants. Because testing for these autoantibodies is technically simple and inexpensive, pre-emptive screening of individuals with exposure risk, including livestock farm workers and veterinarians, could support targeted preventive measures. In the context of the ongoing H5N1 panzootic with dozens of confirmed human infections, identifying individuals with compromised IFN-I immunity may help reduce human infections and, consequently, opportunities for viral adaptation toward efficient human transmission.

## Introduction

Infection of humans with influenza A viruses (IAVs) from avian species pose a continuous threat to the human population. Although such zoonotic transmissions are rare, human infections with avian subtypes, particularly H5N1 and H7N9, have been associated with case fatality rates of 40–50%, underscoring the importance of understanding the determinants of cross-species transmission.^1^, ^2^, ^3^ The recent global spread of avian H5N1 clade 2.3.4.4b, with extensive outbreaks among birds and mammals and sporadic human infections, has renewed concern over pandemic potential.^4^

The detailed surveillance of H7N9 epidemics in China provided a unique setting to investigate mechanisms that facilitate zoonotic transmission. Between 2013 and 2017, five epidemic waves resulted in >1500 laboratory-confirmed cases with an overall case fatality rate of ∼39% among reported cases.^2^^,^^5^ Population-level seroepidemiological studies indicate that human infections are rare, with meta-analyses estimating seroprevalence at approximately 0.02–0.08% in the general population, depending on study design and serological criteria. Although exposure to poultry represents the main risk factor for infection, seroprevalence among poultry workers remains low (approximately 0.1–0.3%), and only about 7% of laboratory-confirmed H7N9 cases occurred in this group.^5^, ^6^, ^7^ These findings suggest that additional host-specific factors may contribute to H7N9 susceptibility. Several studies have identified human genetic variants leading to increased risk of H7N9 infection, including variations in genes involved in the type I interferon (IFN-I) system.^8^^,^^9^ In a whole-genome sequencing study of 217 H7N9 cases and 116 poultry worker controls, we previously identified a deficiency in the IFN-regulated antiviral factor MxA in 6.5% of patients with H7N9 infection,^10^ highlighting the pivotal role of the IFN-I system in maintaining the avian-human species barrier.

IFN-Is are critical mediators of the innate antiviral immune response, and genetic deficiencies of IFN-I induction and signalling can underlie severe viral illness.^11^ In addition to inborn errors of immunity, pre-existing autoantibodies neutralising IFN-I have been recognised as risk factors for severe disease outcomes and fatality. Such autoantibodies were found to underlie approximately 15% of critical COVID-19 pneumonia and 20% of fatal COVID-19 cases.^12^, ^13^, ^14^ In critically ill patients with COVID-19, IFN-I-neutralising autoantibodies were also identified as a risk factor for herpesvirus reactivation.^15^ They have further been detected in 25% of hospitalised patients with Middle East respiratory syndrome (MERS), in cases of severe adverse reactions to yellow fever vaccination, in 40% of West Nile virus (WNV) encephalitis, in 10% of severe tick-borne encephalitis (TBE), in rare severe arboviral diseases including infections with Powassan, Usutu, and Ross River virus, and in approximately 5% of patients with life-threatening pneumonia following seasonal IAV infection.^16^, ^17^, ^18^, ^19^, ^20^, ^21^ Most recently, IFN-I-neutralising autoantibodies were identified in a single fatal case of avian H5N1 pneumonia.^22^ Collectively, these findings suggest that autoantibodies against IFN-I increase the risk for severe viral illness by compromising innate antiviral defences.

Based on this evidence, we hypothesised that anti-IFN-I autoantibodies may predispose humans to infection with avian IAVs. To test this hypothesis, we analysed the prevalence and functional activity of anti-IFN-I autoantibodies in 199 patients with H7N9 infection and 531 healthy, uninfected controls.

## Methods

### Study design and participants

In this observational case–control study, sera from patients with H7N9 infection and healthy, uninfected individuals were tested for the presence of autoantibodies neutralising IFN-I and associations between age, sex, H7N9 case status, case fatality, and autoantibody positivity were assessed (see Supplementary Fig. S1 for study design).

During the five H7N9 epidemics between 2013 and 2017, we collected blood samples and throat swabs from Han Chinese patients with H7N9 infection, healthy poultry workers and close contacts. Once H7N9 infection was laboratory-confirmed in a patient, serum samples and throat swabs from close contacts and epidemiologically linked poultry workers were collected through the influenza surveillance network of the local China CDC. Poultry workers were defined as individuals involved in culling or handling poultry at the market where the index patient was exposed. Close contacts were defined as persons with close physical contact to the symptomatic patient, including healthcare workers, family and household members, and visitors.^10^^,^^23^ According to the diagnostic and treatment protocol for human infections with H7N9 (https://www.nhc.gov.cn/yjb/bmdt/201304/15f4045860954beaa2686a7aedadd88b.shtml), swabs were initially screened for influenza A and B viruses using real-time RT-PCR. IAV-positive samples underwent further testing for H7N9. Positive cases were confirmed by real-time RT-PCR at local CDC facilities. All participants in the control groups tested negative for H7N9. The demographic characteristics of the study participants and sample collection dates after disease onset are detailed in Supplementary Tables S1 and S2, including information about missing data. Sex was self-reported by study participants. The geographic distribution of samples across provinces is summarised in Supplementary Table S3. All patients enrolled in this study were hospitalised. In the Chinese influenza surveillance system patients with suspected H7N9 infection are identified through influenza-like illness and admitted for clinical management of acute respiratory disease. Isolation is implemented within the hospital setting rather than serving as the primary reason for admission. However, apart from disease outcome, we had no access to additional clinical information. Detailed epidemiological and clinical data on the majority of reported H7N9 cases from the five epidemic waves have been published elsewhere.^5^

### Ethics

Written informed consent was obtained from all study participants. The study was approved by the Ethics Committee of the National Institute for Viral Disease Control and Prevention, Chinese Centre for Disease Control and Prevention (ethics vote numbers: IVDC2014-020, IVDC2015-028, IVDC2016-018, and IVDC2017-030), and was conducted in accordance with the principles of the Declaration of Helsinki.

### Cells

A549 (ATCC CRM-CCL-185, RRID:CVCL_0023) and HEK293T (human embryonic kidney cells; ATCC CRL-3216, RRID:CVCL_0063) cells were cultured in Dulbecco's modified Eagle medium (DMEM; ThermoFisher, 11995073), supplemented with Penicillin-Streptomycin (36 U/ml Pen, 36 μg/ml Strep, ThermoFisher, 15140122), and 10% foetal bovine serum in a 37 °C incubator with 5% CO_2_. These cell lines were confirmed to be mycoplasma-free using a commercially available PCR-based assay. However, they have not been authenticated by short tandem repeat (STR) profiling.

### Multiplex bead-based assay

To detect IFN-I-binding autoantibodies in serum samples, we employed a previously established multiplex bead-based immunoassay.^15^ Distinct magnetic beads (MagPlex-C Microspheres, Luminex) were coupled to recombinant human IFNα_2_ (Novus Biologicals, NBP2-34971), IFNβ_1b_ (PBL Assay Science, 11420-1) or IFNω (Novus Biologicals, NBP2-35893). Human albumin-coupled beads served as a control for non-specific binding (albumin: Sigma–Aldrich, A9511). Bead coupling was performed at a concentration of 10 μg antigen per 10^6^ beads. Coupling efficiency was evaluated by incubating the coated beads with monoclonal mouse antibodies against IFNα_2_, IFNβ_1b_, or IFNω (Novus Biologicals, NB100-2479, RRID:AB_10003016; PBL Assay Science, 21465-1, RRID:AB_387836; Novus Biologicals, NBP3-06154, RRID:AB_3542867), followed by detection with phycoerythrin (PE)-conjugated anti-mouse IgG (dilution 1:500; BioLegend, 405307, RRID:AB_315010) (Supplementary Fig. S2). For detection of human anti-IFN autoantibodies, serum samples were diluted 1:100 in PBS containing 1% bovine serum albumin (BSA) and incubated with equal parts of the four differently coated beads for 1 h at room temperature in 96-well plates. PE-labelled goat anti-human IgG (dilution 1:500; Southern-Biotech, 2040–09, RRID:AB_2795648) was used to detect autoantibodies bound to the beads (1 h incubation at room temperature). Beads were washed twice with PBS +1% BSA after each incubation step and analysed on a Luminex 200 system (RRID:SCR_028024). A minimum of 50 events per bead type were acquired per sample. For each IFN, the median fluorescence intensity (MFI) was calculated and normalised against the MFI of albumin-coated control beads to correct for background. We used 8 sera without anti-IFN-I autoantibodies as negative controls which were included on each 96-well plate measured to account for variability between measurements. Because the distribution of normalised MFI values approximated a log-normal distribution (Supplementary Fig. S3), data were log-transformed prior to further analysis. Z scores were calculated based on the mean and standard deviation of the 8 negative controls for each 96-well plate. Owing to the limited availability of patient serum samples, the multiplex bead-based assay was performed without technical replicates. Samples with a Z score >7 were classified as positive for IFN-I-binding antibodies. To minimise false-positive detection and ensure high specificity, we applied a very stringent cutoff for the identification of IFN-binding sera. Given the limited amount of serum available from the patients, our aim was to identify samples with a high likelihood of neutralising activity rather than merely weak or nonspecific binding. Based on previous reports indicating an approximate prevalence of 1–3% neutralising anti-IFN-I autoantibodies in the general population (age-dependent),^12^ we selected a threshold that minimised the likely inclusion of non-neutralising samples.

### Luciferase reporter assay

To assess the capacity of autoantibody-positive sera to neutralise IFN-I signalling, we performed a luciferase-based reporter assay adapted from Busnadiego et al.^15^ HEK293T cells were seeded in 96-well plates (2 × 10^4^ cells per well) and reverse-transfected using FuGene HD (Promega, E2311) with a total of 34 ng plasmid DNA. This included 10 ng of pGL4.45[luc2P/ISRE/Hygro] (Promega, E4141), encoding firefly luciferase under the control of five copies of the IFN-stimulated response element (ISRE) promoter, 4 ng of pRL-SV40 (Promega, E2231), constitutively expressing *Renilla* luciferase to normalise transfection efficiency, and 20 ng of empty vector (pUC18, RRID:Addgene_50004). Twenty-four hours post-transfection, mixtures of recombinant IFN-I and serum samples were added to the cells. Serum was diluted 1:50 in DMEM supplemented with 10% FCS and penicillin/streptomycin, and pre-incubated for 1 h at room temperature with one of the following IFN concentrations: (i) IFNα_2_ (Novus Biologicals, NBP2-34971) at 10 ng/ml or 0.5 ng/ml; (ii) IFNβ_1b_ (PBL Assay Science, 11420-1) at 1 ng/ml or 0.25 ng/ml; (iii) IFN-ω (Novus Biologicals, NBP2-35893) at 10 ng/ml or 0.2 ng/ml. After incubation for 24 h at 37 °C, cells were lysed for 30 min at room temperature, and luciferase activities were measured using the Dual-Luciferase Reporter Assay (Promega, E1960) and a GloMax plate reader (Promega, RRID:SCR_023469), following the manufacturer’s protocol. As a background control, transfected cells were treated with serum, but without IFN for each sample. Four autoantibody-negative sera were included on each 96-well plate as negative controls (negative pool). After normalising firefly to *Renilla* luciferase activities, the resulting values were normalised first, to mock-treated cells receiving serum only (no IFN), and second, to the mean of relative luciferase activities of the negative controls. Each serum sample was tested in two independent experiments and classified as neutralising if the mean of the normalised luciferase activities dropped below 25% of the negative control pool.

### Virus neutralisation assay

To test whether sera neutralising IFN-I signalling in the luciferase-based reporter assay also interfered with IFN-I-mediated antiviral activity, infection experiments were performed by adapting a previously published protocol from Zhang et al.^21^ A549 cells were seeded into 96-well plates at a density of 2 × 10^4^ cells per well and cultivated overnight. Serum samples or a commercial monoclonal anti-IFNα_2_ antibody (positive control from Novus Biologicals, NB100-2479, RRID:AB_10003016) were serially diluted in 10-fold steps, starting from a concentration of 2 × 10^−2^. Diluted samples were incubated for 1 h at 37 °C with 5 ng/ml recombinant IFNα_2_ (Novus Biologicals, NBP2-34971). After removing the culture medium, cells were treated with the pre-incubated serum/antibody-IFNα_2_ mixtures overnight. The mixtures were then removed, and cells were washed three times with PBS to eliminate any possible IAV neutralising antibodies present in the serum samples. Cells were subsequently infected with GFP-expressing PR8 (H1N1; A/Puerto Rico/8/1934) at a multiplicity of infection (MOI) of 1. At 7 h post-infection cells were fixed with 4% formaldehyde, washed twice with PBS, and stained with DAPI. The proportion of infected (GFP-positive) cells was determined using a Keyence BZ-X800 Fluorescence Microscope (RRID:SCR_023617). Infection rates were normalised to infected wells that received no serum, control antibody, or IFNα_2_ treatment. Samples with sufficient material (12 out of 19 neutralising samples) were analysed in two independent experiments.

### Statistics

Study size was determined by sample availability, not by a priori power calculation. We performed a complete case analysis, excluding only one patient with missing age and sex data.

To assess the association between age, sex, and the presence of neutralising autoantibodies, we applied Firth’s penalised logistic regression,^24^ fitting separate models for H7N9 cases and healthy controls. The outcome was the presence of neutralising autoantibodies; predictors were age (continuous), sex (binary), and their interaction. Predicted probabilities were generated for males and females across ages 20–80 years.

To examine the association between neutralising autoantibody responses and H7N9 case status, we fitted additional Firth’s penalised logistic regression models to reduce small-sample bias and address data separation. Comparisons were made between cases and all controls combined as well as each control group individually. Models included age and sex as covariates, and predictors assessed autoantibodies neutralising any IFN-I, IFNα_2_ and/or IFNω, or IFNα_2_ or IFNω.

Robustness of associations between IFN-I-neutralising autoantibodies and case status was evaluated using E-values.^25^ E-values quantify the minimum strength of association (on the risk ratio scale) that an unmeasured confounder would need to have with both the exposure and outcome, conditional on the measured covariates, to fully explain away the observed association.

To investigate factors associated with fatality among H7N9 cases, we fitted a multivariable logistic regression model with death as the outcome and age, sex, and the presence of neutralising autoantibodies as predictors.

We used the statistical software R version 4.3.3 (RRID:SCR_001905) and the R packages EValue, logistf and sjPlot. The significance level was set to p = 0.05.

### Screening for gene variants associated with autoimmune disease

In our previous study, we conducted whole-genome sequencing (WGS) on 217 patients with H7N9 infection and 116 healthy poultry workers as controls.^10^ Briefly, sequencing reads were aligned to GRCH 37.p10 using BWA (0.75) software. Single-nucleotide variants (SNVs) and small insertions/deletions (indels) were identified and genotyped by GATK following the best-practice recommended pipeline. Then, the variants were annotated by ANNOVAR (20250302). Of the 38 patients and 2 poultry workers with neutralising anti-IFN-I autoantibodies detected here, WGS data from our previous study were available for 25 patients and one poultry worker and were screened for coding low frequency and rare variations in genes related to the development of anti-IFN-I autoantibodies.

### Role of funders

The funders of the study had no role in study design, data collection, data analysis, data interpretation, or writing of the report, nor in the decision to submit the manuscript for publication.

## Results

### Study population characteristics

**Fig. 1:**
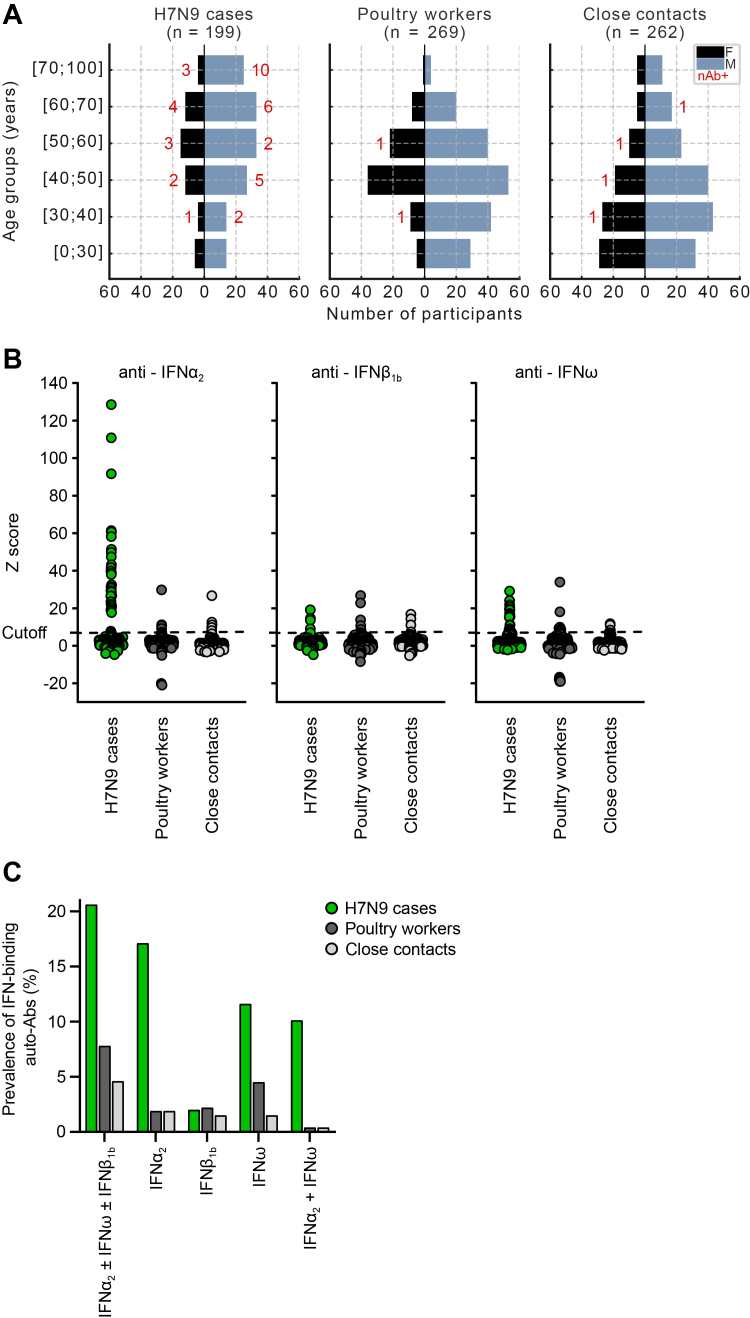
**Autoantibodies binding to IFNα_2_, IFNβ_1b_ and IFNω in patients with H7N9 infection and healthy controls. (A)** Age and sex distribution of the three study groups. For each age group, the number of individuals positive for autoantibodies neutralising at least one tested IFN-I (IFNα_2_, IFNβ_1b_, IFNω) at low concentrations is indicated in red. M, male; F, female; nAb+, positive for IFN-I-neutralising autoantibodies. **(B)** Detection of IgG autoantibodies binding to IFNα_2_, IFNβ_1b_ or IFNω in serum samples by multiplex bead-based assay. Samples with a Z-score >7 were considered positive for IFN-I-binding autoantibodies. Measurements were performed without technical replicates because of limited sample availability. **(C)** Prevalence of IFN-I-binding autoantibodies by IFN type and study group. IFNα_2_ ± IFNβ_1b_ ± IFNω, positive for autoantibodies binding to at least one of the tested IFN-I; IFNα_2_ + IFNω, positive for autoantibodies binding to both IFNα_2_ and IFNω.

We enrolled 199 patients with confirmed H7N9 infection as well as 269 poultry workers and 262 close contacts as healthy controls (Supplementary Tables S1 and S2, Fig. 1A). Both control groups remained uninfected despite exposure to poultry or close contact with symptomatic index cases. The patient group had a mean age of 53 years (range 1–91), 73.4% were men (146/199) and the case fatality rate was 41.2% (82/199). These demographic and clinical characteristics are consistent with previous reports from the five H7N9 epidemics.^5^ Both control groups were sex- and age-matched to the patients. Poultry workers ranged in age from 7 to 83 years with a mean age of 45 years (69.9% men, 188/269), and close contacts from 18 to 89 years with a mean age of 42 years (63.4% men, 166/262).

### IFN-I-neutralising autoantibodies in 19.1% of patients with H7N9 infection

**Fig. 2:**
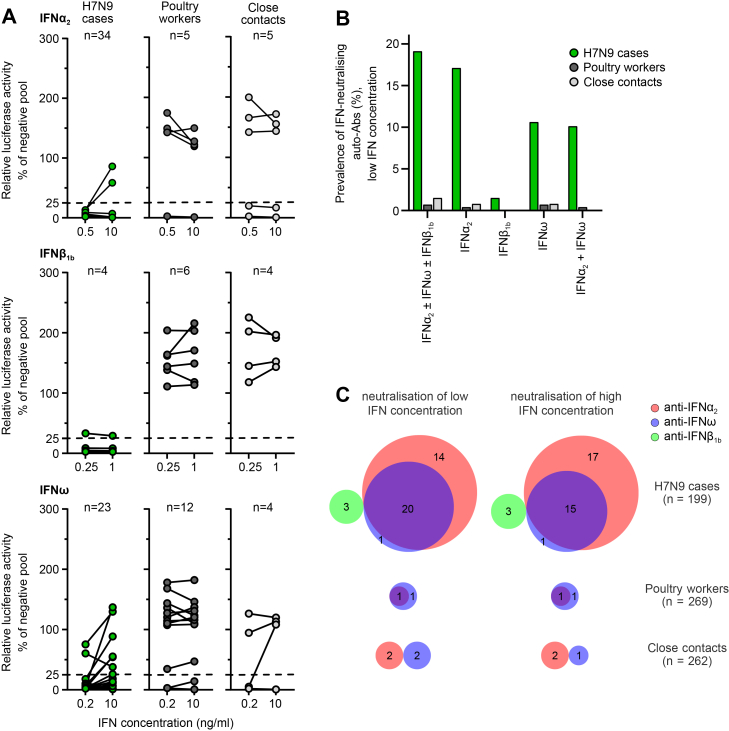
**Autoantibodies neutralising IFN-I in patients with H7N9 infection and healthy controls. (A)** Luciferase-based reporter assay to assess the capacity of autoantibody positive sera to neutralise IFNα_2_ (10 or 0.5 ng/ml), IFNβ_1b_ (1 or 0.25 ng/ml) or IFNω (10 or 0.2 ng/ml). Each sample was tested in biological duplicates and the mean values are shown. Samples were classified as neutralising if the mean of the relative luciferase activities was below 25% (dotted line) of the mean of the negative pool (four autoantibody-negative control sera). All sera positive for IFN-I-binding autoantibodies were tested; numbers are indicated above the graphs. Lines connect measurements of neutralising activity from the same serum sample at low and high IFN concentrations. **(B)** Prevalence of autoantibodies neutralising low IFN concentrations (IFNα_2_: 0.5 ng/ml, IFNβ_1b_: 0.25 ng/ml; IFNω: 0.2 ng/ml) by IFN type and study group. IFNα_2_ ± IFNβ_1b_ ± IFNω, positive for autoantibodies neutralising at least one tested IFN-I; IFNα_2_ + IFNω, positive for autoantibodies neutralising both IFNα_2_ and IFNω. **(C)** Area-proportional Venn diagrams illustrating the absolute numbers of samples with autoantibodies neutralising high and low concentrations of IFNα_2_ (10 or 0.5 ng/ml), IFNβ_1b_ (1 or 0.25 ng/ml) or IFNω (10 or 0.2 ng/ml). Venn diagrams were created with BioVenn (https://www.biovenn.nl/index.php).^26^

To initially screen for the presence of IFN-I-specific autoantibodies, we performed a high-throughput, low-volume multiplex bead-based assay detecting IgG binding to representative type I IFNs including IFNα_2_, IFNβ_1b_, and IFNω (Fig. 1B). We identified 20.6% of patients (41/199) with IgG autoantibodies recognising at least one IFN-I. Autoantibodies were less frequent in the control groups. 7.8% of poultry workers (21/269) and 4.6% of close contacts (12/262) were tested positive (Supplementary Table S4, Fig. 1C). To assess the functional relevance of the IFN-binding autoantibodies, we tested the ability of the autoantibody-positive sera to block IFN-I-signalling in a luciferase-based reporter assay (Fig. 2A). HEK293T cells expressing firefly luciferase under the control of the human ISRE were stimulated with low or high concentrations of IFNα_2_ (0.5 or 10 ng/ml), IFNβ_1b_ (0.25 or 1 ng/ml) or IFNω (0.2 or 10 ng/ml) in the presence of sera diluted 1:50. Sera without detectable IFN-binding were not tested further and classified as non-neutralising (Supplementary Fig. S1). Overall, 19.1% of patients with H7N9 infection (38/199) harboured autoantibodies neutralising at least one IFN-I at low concentration, compared with 0.7% (2/269) in poultry workers and 1.5% (4/262) in close contacts (Fig. 2B, Table 1). Neutralising activity among patients was predominantly directed against IFNα_2_ (34/199). Only three patients had anti-IFNβ_1b_ autoantibodies. Except for one sample, patient sera neutralising IFNω also inhibited IFNα_2_ signalling (20/199 double positives) (Fig. 2C). Of note, most neutralising patient sera also blocked high IFN concentrations (36/199; 18.1%) (Fig. 2A, Table 1). Only two patients, one with autoantibodies against IFNα_2_ alone and one with autoantibodies against IFNα_2_ and IFNω, harboured autoantibodies exclusively neutralising low IFN concentrations. Four patients harboured anti-IFNω autoantibodies unable to neutralise the high IFN concentration, but carried anti-IFNα_2_ autoantibodies with high neutralisation capacity (Supplementary Table S1). Among controls, all but one neutralising serum also blocked high IFN concentrations (Fig. 2A, Table 1). Taken together, these findings demonstrate a high prevalence of potent IFN-I-neutralising autoantibodies in patients with H7N9 infection, that are not found to the same extent in matched control groups.

**Table 1:** Study participants with autoantibodies neutralising IFN-I

	IFN conc.	H7N9 cases n = 199	Poultry workers n = 269	Close contacts n = 262
Total	low	38 (19.1%)	2 (0.7%)	4 (1.5%)
	high	36 (18.1%)	2 (0.7%)	3 (1.1%)
anti-IFNα_2_	0.5 ng/ml	34 (17.1%)	1 (0.4%)	2 (0.8%)
	10 ng/ml	32 (16.1%)	1 (0.4%)	2 (0.8%)
anti-IFNβ_1b_	0.25 ng/ml	3 (1.5%)	0	0
	1 ng/ml	3 (1.5%)	0	0
anti-IFNω	0.2 ng/ml	21 (10.6%)	2 (0.7%)	2 (0.8%)
	10 ng/ml	16 (8.0%)	2 (0.7%)	1 (0.4%)
Double positive anti-IFNα_2_ + anti-IFNω	low	20 (10.1%)	1 (0.4%)	0
	high	15 (7.5%)	1 (0.4%)	0
Double positive anti-IFNα_2_ + anti-IFNβ_1b_	low	0	0	0
	high	0	0	0

Data are n (%). IFN conc. = interferon concentration.

### Age-associated prevalence of IFN-I-neutralising autoantibodies in patients with H7N9 infection

**Fig. 3:**
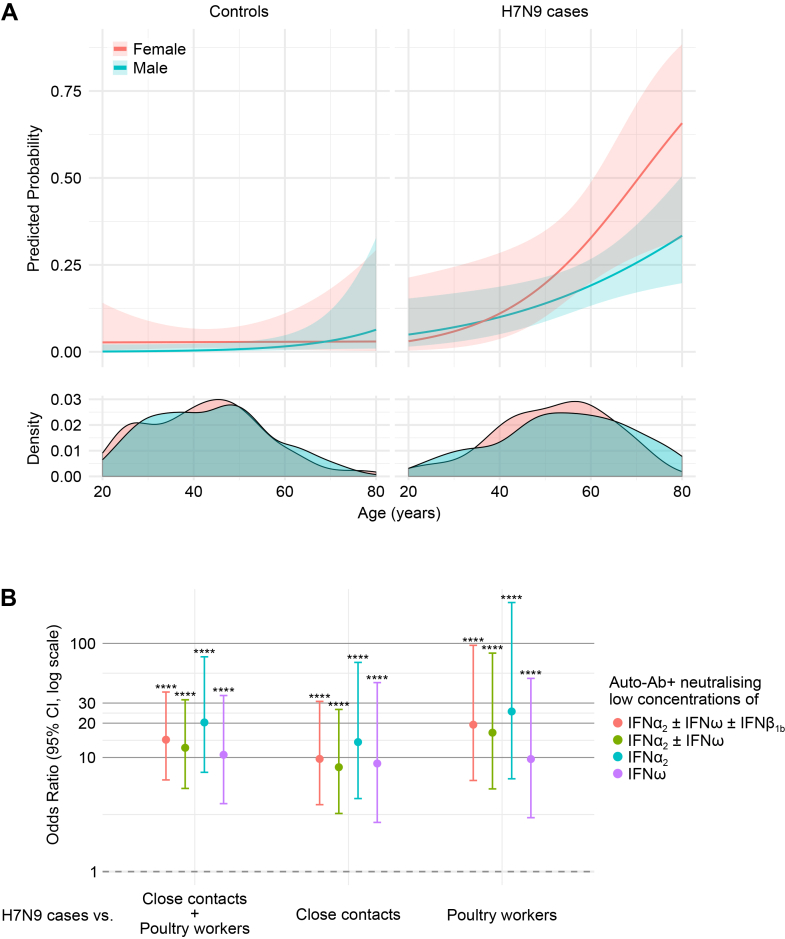
**Association between the presence of IFN-I-neutralising autoantibodies and H7N9 infection. (A)** The association between age, sex and IFN-I-neutralising autoantibodies in patients with H7N9 infection or in the two control groups combined (poultry workers + close contacts) was assessed using Firth's penalised logistic regression. Predicted probabilities for the presence of autoantibodies with 95% confidence intervals (CIs, shaded areas around the curve) are shown across participant age for men and women. To visualise the modelled probabilities in relation to the underlying data, we overlaid sex-specific age density distributions beneath the predicted probability curves. **(B)** Odds ratios (OR) with 95% CIs for the presence of autoantibodies neutralising low IFN concentrations in patients compared to healthy controls, adjusted for age and sex, determined by Firth’s penalised logistic regression models. See also Supplementary Table S6 for the results of the logistic regression analyses and Supplementary Table S7 for unadjusted estimates. IFNα_2_ ± IFNω ± IFNβ_1b_, positive for autoantibodies neutralising at least one of the tested IFN-I; IFNα_2_ ± IFNω, positive for autoantibodies neutralising IFNα_2_ and/or IFNω; ∗∗∗∗, p < 0.0001 (Firth’s penalised logistic regression).

To investigate demographic factors associated with the presence of IFN-neutralising autoantibodies, we analysed the age and sex distribution of individuals whose sera neutralised at least one IFN-I at low concentrations using Firth’s penalised logistic regression (Fig. 3A, Supplementary Table S5). Among H7N9 cases, the autoantibody prevalence was 17.1% in men (25/146) and 24.5% in women (13/53). Although this indicates a higher prevalence among female patients, the association between sex and autoantibody positivity was not statistically significant (odds ratio [OR] for males vs. females = 0.52; 95% confidence interval [CI] 0.23–1.14; p = 0.106) (Fig. 3A, Supplementary Table S5). Autoantibody-positive patients ranged in age from 31 to 88 years, with about one-third (13/38, 34.2%) aged ≥70 years. Among patients ≥70 years old, 44.8% (13/29) had detectable neutralising autoantibodies, compared to only 14.7% (25/170) of those <70 years old. Increasing age was significantly associated with autoantibody positivity (OR = 1.05; 95% CI 1.02–1.07; p = 0.0001) (Fig. 3A, Supplementary Table S5). This age effect was similar in men and women. We did not observe an association between the presence of IFN-I-neutralising autoantibodies and age or sex among healthy controls, although the overall low prevalence of autoantibody-positive individuals in the control groups (6/531 combined poultry workers and close contacts) limits the weight of this conclusion (Fig. 3A). In summary, our data revealed a significant age-dependent increase in the prevalence of IFN-I-neutralising autoantibodies among patients with H7N9 infection, with a trend toward higher frequency in female patients.

### Association of IFN-I-neutralising autoantibodies with increased risk of H7N9 infection

To determine whether the presence of IFN-I-neutralising autoantibodies is associated with H7N9 infection, we compared their prevalence between patients and the two healthy control groups using sex- and age-adjusted Firth’s penalised logistic regression. Autoantibodies neutralising any tested IFN-I at low concentrations were significantly enriched in patients relative to each control group individually and combined (Fig. 3B, Supplementary Table S6). Depending on autoantibody type and control group, their presence was associated with 8.2- to 25.3-fold higher odds of H7N9 infection (Supplementary Table S6; see Supplementary Table S7 for unadjusted estimates). IFNα_2_-neutralising autoantibodies were the strongest predictor of H7N9 infection. To assess robustness, we calculated E-values which ranged from 18.9 to 38.3 when comparing the prevalence of any IFN-I-neutralising autoantibody between cases and the control groups (Supplementary Table S6). These large E-values indicate that substantial unmeasured confounding would be required to explain away the observed association between H7N9 infection and the presence of IFN-neutralising autoantibodies. Autoantibodies neutralising at least one IFN-I at high concentrations were likewise significantly more frequent in patients with H7N9 infection (Supplementary Table S8). Despite this strong association with H7N9 infection, we found no statistically significant relationship between autoantibody positivity and disease outcome. In a multivariable logistic regression model, the presence of neutralising autoantibodies was not associated with increased fatality (OR = 1.16; 95% CI 0.55–2.43; p = 0.699) (Supplementary Table S9). Among the 38 autoantibody-positive patients, 47.4% died, compared to an overall case fatality rate of 41.2%. Age and sex were also not associated with fatal outcome (Supplementary Table S9). Taken together, these findings demonstrate a robust and statistically significant association between IFN-I-neutralising autoantibodies and H7N9 infection, regardless of control group or autoantibody type.

### Autoantibody-positive sera neutralise IFNα-mediated antiviral activity in cell culture

**Fig. 4:**
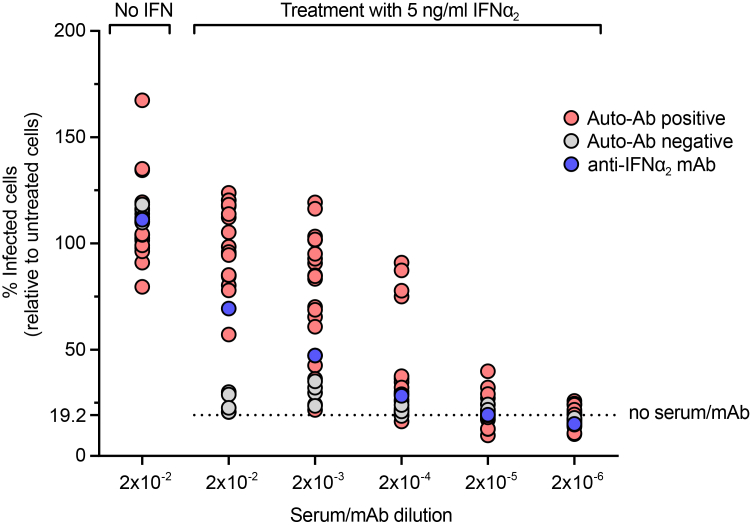
**Neutralising sera block the antiviral effect of IFNα_2_ in cell culture infected with IAV.** Antiviral activity of IFNα_2_ (5 ng/ml) against IAV (PR8-GFP, MOI 1) alone or in the presence of serially diluted IFN-I-neutralising sera (n = 19), autoantibody-negative sera (n = 4), or a monoclonal anti-IFNα_2_ antibody in A549 cells. Infection rates (GFP^+^/DAPI^+^ cells) at 7 h post-infection were normalised to untreated, infected cells. The dotted line indicates the reduction of infected cells after IFN treatment alone. If possible, the mean of two independent experiments is shown. Sufficient material was available for 12 out of 19 samples.

We next investigated whether autoantibodies that blocked IFN-I signalling in the luciferase-based neutralisation assay also impaired the antiviral activity of IFN-I in a cellular infection model. A549 epithelial cells were treated with IFNα_2_ pre-incubated with or without sera prior to infection with a GFP-expressing IAV and the proportion of infected (GFP-positive) cells was quantified. Pre-treatment with 5 ng/ml IFNα_2_ strongly suppressed infection, reducing the proportion of infected cells to 19.2% compared to untreated, infected cells (Fig. 4). Sera from a representative subset of individuals carrying neutralising anti-IFN-I autoantibodies, including 1 poultry worker, 2 close contacts, and 16 patients, substantially diminished or completely abolished this antiviral effect when diluted 1:200. Most human sera showed stronger neutralising activity than the positive control, an anti-IFNα monoclonal mouse antibody. In contrast, control sera without anti-IFN-I autoantibodies did not interfere with IFNα_2_-mediated antiviral activity at the same dilution. The weakest inhibition of antiviral restriction was observed in a patient serum that only neutralised low IFN concentrations in the luciferase-based neutralisation assay, and reduced the antiviral effect by only ∼50% in the infection assay. Notably, four patient sera effectively inhibited the antiviral activity of IFNα_2_ even at a dilution of 1:20,000, suggesting a high antibody titre. As expected, the neutralising effect of both human sera and the monoclonal antibody declined with increasing dilution. These findings show that the detected anti-IFN-I autoantibodies can neutralise the IFN-I-mediated antiviral response in cell culture, supporting that they may similarly enhance H7N9 replication and susceptibility.

### No known genetic predisposition to IFN-I autoimmunity in autoantibody-positive individuals

Autoimmune responses against IFN-I are typically prevented by central tolerance. In several monogenic autoimmune syndromes, genetic variations disrupting self-tolerance pathways have been linked to anti-IFN autoantibody development.^27^ To investigate whether such mechanisms might underlie autoantibody formation in our study groups, we analysed WGS data available from 25 of 38 (65.8%) autoantibody-positive patients and from one of two (50%) autoantibody-positive poultry workers. These data derived from our previous study on host genetic susceptibility to H7N9 infection.^10^ We screened for low-frequency and rare variations in the coding region of genes previously associated with the development of anti-IFN-I autoantibodies. This included genes involved in thymic negative selection via regulating expression of tissue-specific self-antigens in medullary thymic epithelial cells: AIRE, NFKB2, MAP3K14 (NIK), IKBKG (NEMO) and RELB.^28^^,^^29^ Additionally, we analysed genes for which deficiencies also have been reported in patients with anti-IFN-I autoantibodies, including RAG1, RAG2, FOXP3, IKZF2, CTLA4 and PTCRA.^28^^,^^29^ None of the autoantibody-positive individuals in our study harboured known deficiencies or previously undescribed low-frequency or rare variations in these genes that could plausibly account for a genetically determined loss of tolerance to IFN-I.

## Discussion

In this study, we show that IFN-I-neutralising autoantibodies are considerably enriched in patients with H7N9 infection compared to uninfected, but exposed control groups. Nearly one-fifth (19.1%) carried autoantibodies neutralising at least one IFN-I at low concentrations, with the majority also neutralising high IFN-I levels (18.1%). These autoantibodies were directed predominantly against IFNα_2_ alone or against IFNα_2_ and IFNω, but rarely against IFNβ_1b_. This pattern aligns with observations in anti-IFN-I autoantibody-positive patients with severe viral infections, including critical seasonal influenza and COVID-19 pneumonia, WNV disease, and severe TBE.^13^^,^^18^^,^^19^^,^^21^ Importantly, the prevalence of IFN-neutralising autoantibodies among Chinese healthy controls was much lower, consistent with previous population-level estimates. Among individuals younger than 70 years, 1.2% (6/509; poultry workers and close contacts combined) were positive for neutralising autoantibodies against IFNα_2_ and/or IFNω, matching the prevalence of ∼1.1% reported in a group of more than 10,000 healthy individuals (<70 years, neutralising 100 pg/ml IFN-I).^12^

Because the available serum volume was limited, we applied a stringent positivity threshold (Z score >7) in the binding assay to minimise false positives and restricted neutralisation testing to binding-positive samples. As a result, the true prevalence of neutralising autoantibodies in our study groups may be underestimated.

In patients, the prevalence of IFN-neutralising autoantibodies increased markedly with age, reaching 44.8% among individuals aged ≥ 70 years. This age-dependency has also been reported for critical cases of influenza and COVID-19, and WNV disease, as well as in large samples from the general population, where approximately 4% of individuals over 70 years, but only ∼1% of younger individuals harbour such autoantibodies.^12^^,^^18^^,^^21^^,^^30^ We did not observe this age association in our healthy Chinese control groups, likely due to the very low number of autoantibody-positive individuals. The emergence of these autoantibodies in late adulthood has been linked to age-related thymic involution, and longitudinal cohort data suggest a median age of onset around 63 years.^28^^,^^30^ Previous studies of critical seasonal influenza and COVID-19 also reported a higher prevalence of IFN-I-neutralising autoantibodies in men, whereas we did not observe such a sex association among patients with H7N9 infection.^12^^,^^13^^,^^21^

Although advanced age is a known risk factor for mortality in H7N9 infection,^5^ and despite the increase in autoantibody prevalence with age among H7N9 cases, we did not observe an association between the presence of IFN-I-neutralising autoantibodies and fatal outcome. While fatality rates among H7N9 cases in our study were higher in older patients, the observed differences did not achieve statistical significance. Because clinical data, such as ICU admission, duration of hospitalisation, or requirement for mechanical ventilation, were not available for the H7N9 cases included in this study, we could not assess whether neutralising anti-IFN-I autoantibodies correlate with disease course and severity after H7N9 infection as shown for other viral infections. However, all patients included in this study were hospitalised and previously published epidemiological and clinical data of 1220 patients with H7N9 infection across the five epidemics indicate that 55% of patients required ICU admission and 46% mechanical ventilation.^5^ Thus, a bias toward severe cases in our study population cannot be excluded and may have obscured potential associations with fatality.

Instead, our findings indicate that anti-IFN-I autoantibodies substantially increase susceptibility to H7N9 infection. Depending on antibody specificity and reference group, individuals with neutralising autoantibodies had an 8- to 25-fold higher risk of infection.

The size of the case group was limited, reflecting the overall small number of confirmed H7N9 cases. As study size was determined by the availability of serum samples rather than a priori power calculation, Firth’s penalised logistic regression was applied to mitigate bias related to small sample size and potential data separation. Due to the epidemiological characteristics of the H7N9 epidemics, adjustments for age and sex were required. However, since detailed clinical information, including comorbidities and disease severity, was not available, residual confounding from such covariates cannot be excluded. Although some uncertainty in the effect estimates remains, the magnitude of the observed associations, together with their biological plausibility, support a causal contribution of anti-IFN-I autoantibodies to H7N9 susceptibility. This is further strengthened by the large E-values, suggesting that the observed associations are highly robust to unmeasured confounding.

Neither serum samples prior to H7N9 infection nor from multiple time points after disease onset were available to exclude that these autoantibodies arose during H7N9 infection. It has been speculated that some viral infection conditions might trigger the development of anti-IFN-I autoantibodies.^28^ However, multiple studies with longitudinal data from patients infected with TBEV, WNV and seasonal IAV indicate that IFN-I autoantibodies rarely arise *de novo* after acute infection.^18^^,^^19^^,^^21^ Furthermore, zoonotic H7N9 infection induces relatively low IFN-I levels compared to seasonal IAV or H5N1,^31^^,^^32^ making infection-triggered autoantibody development less likely. Moreover, several autoantibody-positive patients in our study had blood drawn within 1–3 days of symptom onset, and all autoantibodies were high-affinity IgG rather than IgM (Supplementary Table S1). Together, these observations strongly indicate that the detected autoantibodies were pre-existing.

Genetic factors may also contribute to the development of neutralising anti-IFN-I autoantibodies, but we did not identify pathogenic variants known to underlie IFN-I autoimmunity in autoantibody-positive study participants with available WGS data. The WGS data analysed here were acquired in our earlier study identifying the antiviral ISG *MX1* as a susceptibility gene for H7N9 infection, with inactive MxA variants present in 6.5% of patients.^10^ Among the 133 patients included in both studies, including 13 carriers of *MX1* variants (Supplementary Table S1), only one individual harboured both an inactive *MX1* variant and neutralising anti-IFN-I autoantibodies, indicating minimal overlap between these two susceptibility factors. Taken together, the combined data suggest that defects in the IFN-I system, either inborn or autoimmune, may account for up to 25% of H7N9 infections.

These findings underscore the central role of IFN-I immunity in maintaining the avian-human interspecies barrier and preventing zoonotic IAV infections. In the context of the ongoing global H5N1 panzootic, this has direct relevance. Avian H5N1 clade 2.3.4.4b isolates from mammals are sensitive to MxA antiviral activity and therefore to IFN-I-mediated restriction.^33^ Screening for IFN-I-neutralising autoantibodies in exposed individuals may offer a feasible and cost-effective strategy for risk stratification and help guide targeted preventive interventions for high-risk groups, including prioritisation for vaccination or enhanced monitoring.

## Contributors

YC, TB and DW enrolled participants and collected blood samples. YC and TB organised clinical sample data and performed sample screening. JA, TJ and LY performed experimental assays. JA, TJ, JF and LG verified and analysed the experimental data. MW did the statistical analyses. KG, RK, BGH, and GK established the multiplex bead-based and the luciferase reporter assays. JA, MW, JF, and LG created the figures. LG wrote the first draft of the report. YC and LG supervised the study. YC, YS and LG accessed and verified the data. YC, MS, YS, and LG conceived of the study. All authors critically revised the manuscript, and read and approved the final version. All authors had full access to all the data in the study and accept responsibility for the decision to submit for publication.

## Data sharing statement

All data supporting the findings of this study are included in the article and appendix. Raw experimental data are available from YC (chenyk@szu.edu.cn) and LG (laura.graf@uniklinik-freiburg.de) upon request. Code is available on GitHub (https://github.com/jonas-fuchs/distributionTester). WGS data analysed in this study were published previously.^10^ Due to patient privacy, de-identified WGS data are available through controlled access via the China National GeneBank DataBase (CNGBdb) (https://db.cngb.org/search/project/CNP0001997/) for research and non-commercial purposes only upon approval by DW and YS in accordance with the Regulations on the Management of Human Genetic Resources of China.

## Declaration of interests

JA declares that he received a GfV Travel Grant to present this work at the 34th Annual Meeting of the Society of Virology (GfV). MW declares that he received financial support from ELSEVIER to visit the CMI Editor meeting and the ESCMID Global conference. All other authors declare no conflict of interest.
