# Supplementary material for: Autoantibodies against type I interferons in patients with zoonotic H7N9 influenza: an observational case–control study
Source: eBioMedicine. 2026 Jul 16;130:106387. doi: 10.1016/j.ebiom.2026.106387 (PMC13382596; doi:10.1016/j.ebiom.2026.106387)
Supplement: Supplementary Figs. S1–S3 and Tables S2–S9 [file mmc1.pdf]

Supplementary appendix

Autoantibodies against type I interferons in patients with zoonotic H7N9 influenza: an observational case-control study

Supplementary figures

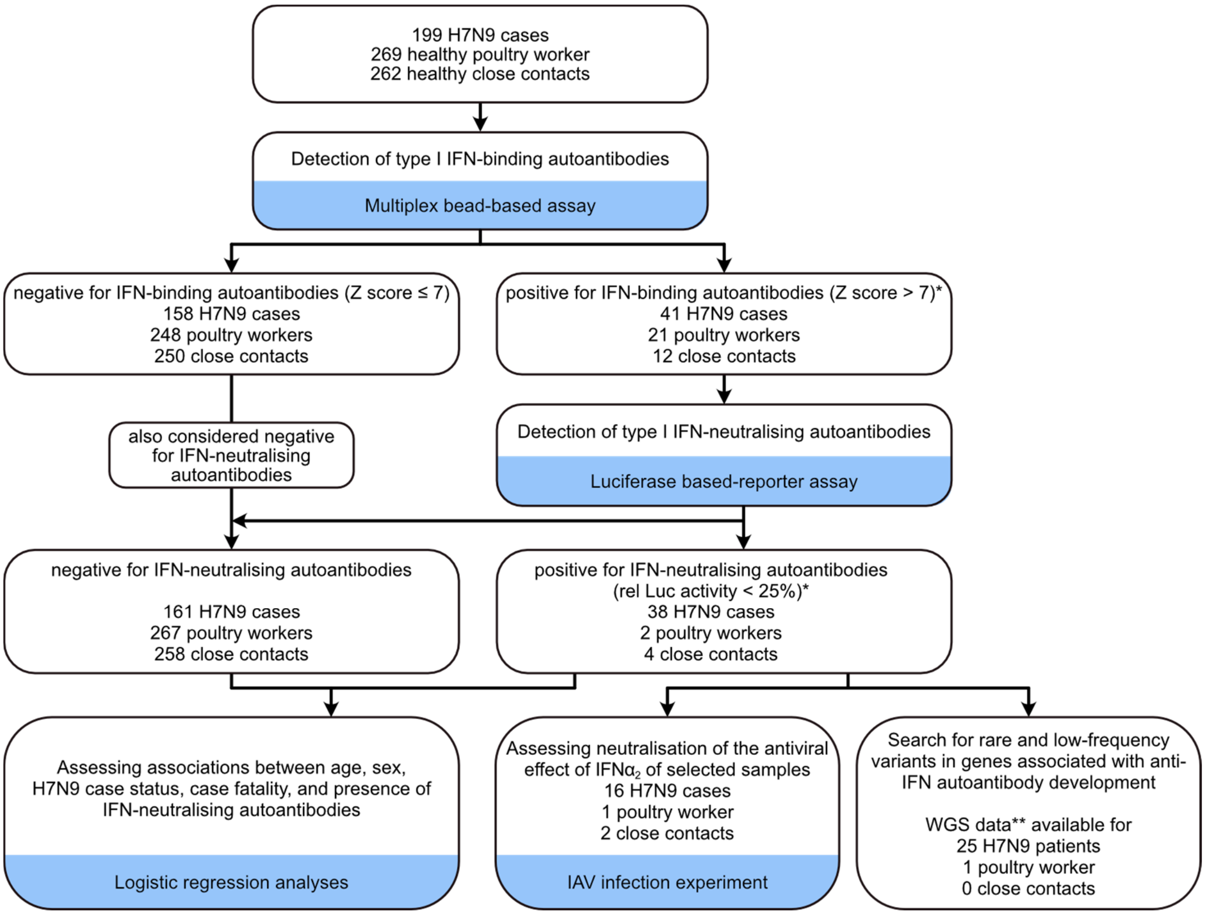

Figure S1: Study design.
The flow chart gives an overview of the performed analyses and the number of samples at each stage. rel Luc activity, percentage of relative luciferase activity compared to negative pool; WGS, whole genome sequencing; *, numbers describe samples positive for autoantibodies binding to at least one of the tested IFN-I or neutralising at least one of the tested IFN-I at low concentrations; **, WGS data from Chen et al., 2021, Science (https://doi.org/10.1126/science.abg5953).

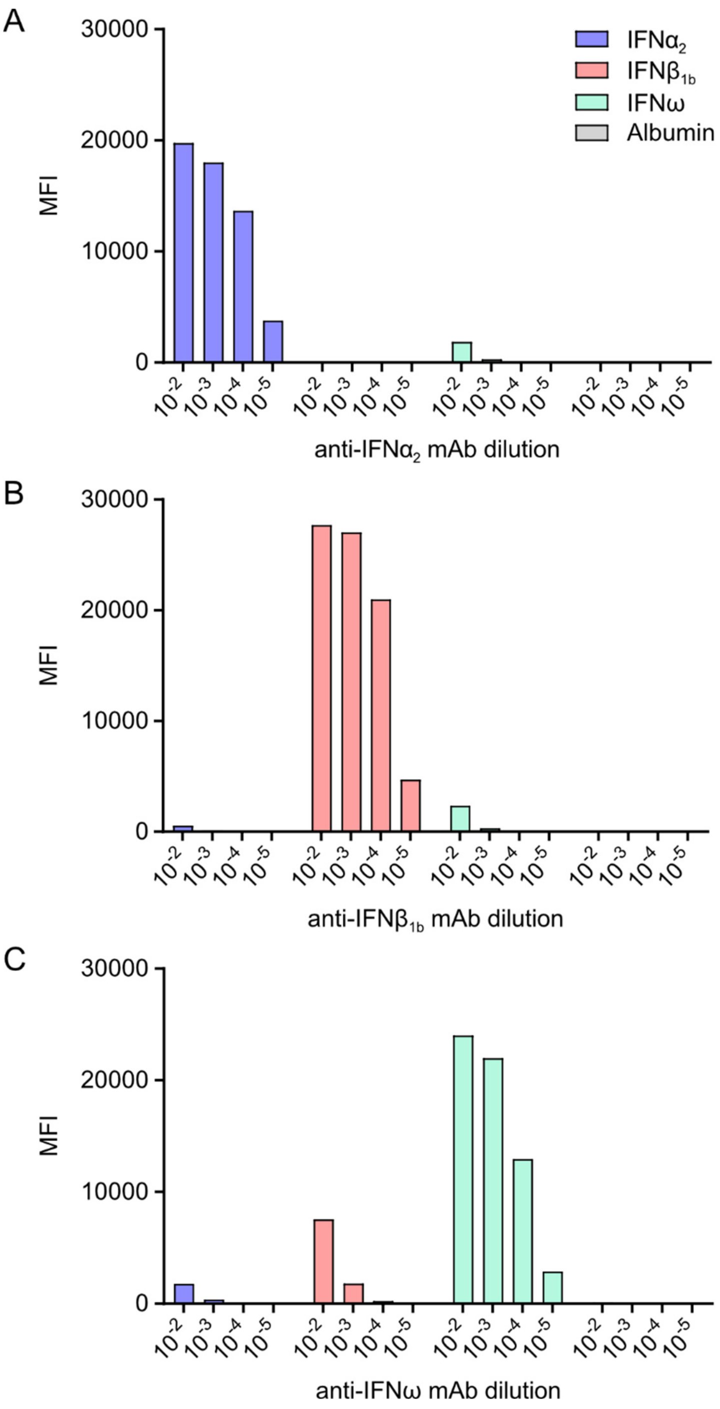

Figure S2: Bead coupling efficiency of human IFNs.
Recombinant human IFNα$_2$, IFNβ$_{1b}$, IFNω or albumin (non-specific binding control) were coupled to magnetic beads for the multiplex bead-based assay. The coated beads were mixed 1:1:1:1 and incubated with serially diluted monoclonal mouse antibodies against IFNα$_2$ **(A)**, IFNβ$_{1b}$ **(B)**, or IFNω **(C)** to assess coupling efficiency. Median fluorescence intensities (MFI) are depicted.

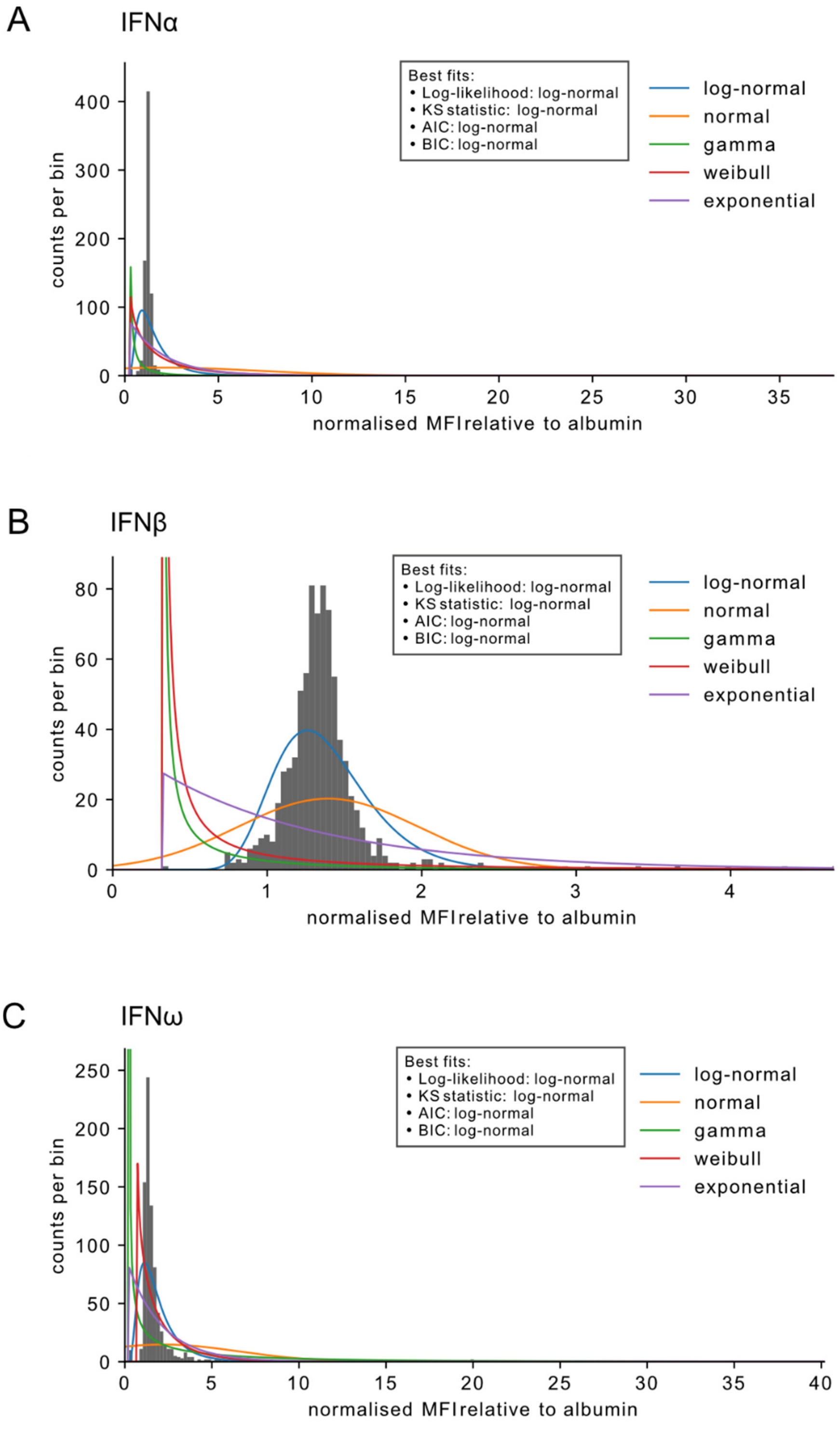

Figure S3: Normalised MFI values from the multiplex bead-based assay are best explained by a log normal distribution.

To evaluate which distribution best fits the albumin-normalised MFI values describing binding to IFNα_2 **(A)**, IFNβ_{1b} **(B)** and FNω **(C)**, different distribution models (normal, log-normal, Weibull, exponential and gamma) were fitted to the respective datasets. The goodness of the individual fits was evaluated by how well the model explains the data (log-likelihood), the maximum deviation between the model and the data (Kolmogorow-Smirnow-Test - KS statistic), and by the Akaike information criterion (AIC) and Bayesian information criterion (BIC). Histograms of the data with the individual curve fits of each distribution are shown. The text box indicates the best fit model according to each statistical test. Figures and statistics were performed with python3.13 using a custom script available at https://github.com/jonas-fuchs/distributionTester.

Supplementary tables

Table S1: Details about study participants and overview of test results

Table is provided as an Excel file.

Table S2: Basic characteristics of study population

	H7N9 cases	Poultry workers	Close contacts
**Sample size**	199	269	262
**Age (years, mean±SD)**	52·8 ± 17·6	44·8 ± 12·2	42·1 ± 14·9
**Age range (years)**	1 - 91	7 - 83	18 - 89
**Male / Female**	146 / 53	188 / 81	166 / 95
**Death / Survival**	82 / 115	··	··

Data are n or mean (SD).

Table S3: Geographic distribution of samples across provinces in China

**Comparison**	**H7N9 cases** n=199	**Close contacts** n=262	**Poultry workers** n=269	**Total** n=730
Anhui Province	14	6	0	20
Beijing	14	11	6	31
Chongqing	7	41	0	48
Fujian Province	35	19	63	117
Gansu Province	1	0	0	1
Guangxi Province	4	4	15	23
Guizhou Province	4	11	0	15
Hebei Province	4	9	0	13
Henan Province	5	22	4	31
Hubei Province	1	8	0	9
Hunan Province	7	19	24	50
Jilin Province	1	0	63	64
Jiangsu Province	55	37	75	167
Jiangxi Province	21	34	0	55
Liaoning Province	3	13	0	16
Shandong Province	14	21	4	39
Shaanxi Province	2	1	0	3
Shanghai	0	1	0	1
Sichuan Province	0	1	8	9
Tianjin	2	0	0	2
Xinjiang Uygur Autonomous Region	5	4	7	16

Data are n.

Table S4: Study participants with autoantibodies binding to IFN-I

	**H7N9 cases** n=199	**Poultry workers** n=269	**Close contacts** n=262
**Total**	41 (20·6%)	21 (7·8%)	12 (4·6%)
**anti-IFNα_2**	34 (17·1%)	5 (1·9%)	5 (1·9%)
**anti-IFNβ_{1b}**	4 (2·0%)	6 (2·2%)	4 (1·5%)
**anti-IFNω**	23 (11·6%)	12 (4·5%)	4 (1·5%)
**Double positive anti-IFNα_2 + anti-IFNω**	20 (10·1%)	1 (0·4%)	1 (0·4%)
**Double positive anti-IFNα_2 + anti-IFNβ_{1b}**	0	1 (0·4%)	0

Data are n (%).

Table S5: Firth's penalised logistic regression to assess the association between age, sex, and the presence of IFN-I-neutralising autoantibodies among H7N9 cases

Predictors	OR [95% CI]	p value
(Intercept)	0.03 [0.01 - 0.13]	<0.0001
Age	1.05 [1.02 - 1.07]	0.0001
Male [Male]	0.52 [0.23 - 1.14]	0.106
Observations	199	
R^2	0.095	

OR, odds ratio; CI, confidence interval

Table S6: Risk of H7N9 infection for individuals positive for IFN-I-neutralising autoantibodies relative to healthy control groups with adjustments for sex and age (low IFN concentrations)

Comparison	Autoantibodies neutralising*	OR [95% CI]	p value	E-Value (lower bound)**
H7N9 vs. CC/PW	$\alpha_2 \pm \omega \pm \beta_{1b}$	14.3 [6.4 - 37.5]	<0.0001	28.1 (12.2)
H7N9 vs. CC	$\alpha_2 \pm \omega \pm \beta_{1b}$	9.7 [3.9 - 31.0]	<0.0001	18.9 (7.1)
H7N9 vs. PW	$\alpha_2 \pm \omega \pm \beta_{1b}$	19.4 [6.3 - 96.2]	<0.0001	38.3 (11.9)
H7N9 vs. CC/PW	$\alpha_2 \pm \omega$	12.2 [5.4 - 32.0]	<0.0001	n.d.
H7N9 vs. CC	$\alpha_2 \pm \omega$	8.2 [3.2 - 26.4]	<0.0001	n.d.
H7N9 vs. PW	$\alpha_2 \pm \omega$	16.5 [5.3 - 82.2]	<0.0001	n.d.
H7N9 vs. CC/PW	α_2	20.3 [7.4 - 76.3]	<0.0001	n.d.
H7N9 vs. CC	α_2	13.6 [4.4 - 68.1]	<0.0001	n.d.
H7N9 vs. PW	α_2	25.3 [6.5 - 228.2]	<0.0001	n.d.
H7N9 vs. CC/PW	ω	10.6 [3.9 - 34.9]	<0.0001	n.d.
H7N9 vs. CC	ω	8.9 [2.7 - 45.3]	<0.0001	n.d.
H7N9 vs. PW	ω	9.7 [3.0 - 49.4]	<0.0001	n.d.

* amount of IFN neutralised, in serum diluted 1:50: 0.5 ng/ml IFNα_2, 0.2 ng/ml IFNω, 0.25 ng/ml IFNβ_{1b}
** An E-value of 28.1, with a lower-bound E-value of 12.2 suggests that an unmeasured confounder would need to be associated with both the exposure and the outcome by a risk ratio of at least 28.1, and by at least 12.2 to reduce the lower confidence limit to the null.
±, and/or; CC, close contacts; PW, poultry workers; CC/PW, healthy control groups combined; OR, odds ratio; CI, confidence interval; n.d., not determined

Table S7: Risk of H7N9 infection for individuals positive for IFN-I-neutralising autoantibodies relative to healthy control groups without adjustments (low IFN concentrations)

Comparison	Autoantibodies neutralising*	OR [95% CI]	p value
H7N9 vs. CC/PW	$\alpha_2 \pm \omega \pm \beta_{1b}$	19.3 [8.8 - 49.5]	<0.0001
H7N9 vs. CC	$\alpha_2 \pm \omega \pm \beta_{1b}$	13.7 [5.6 - 43.0]	<0.0001
H7N9 vs. PW	$\alpha_2 \pm \omega \pm \beta_{1b}$	25.5 [8.4 - 125.4]	<0.0001
H7N9 vs. CC/PW	$\alpha_2 \pm \omega$	17.4 [7.9 - 45.0]	<0.0001
H7N9 vs. CC	$\alpha_2 \pm \omega$	12.4 [5.0 - 39.0]	<0.0001
H7N9 vs. PW	$\alpha_2 \pm \omega$	23.1 [7.6 - 113.7]	<0.0001
H7N9 vs. CC/PW	α_2	31.5 [11.8 - 116.9]	<0.0001
H7N9 vs. CC	α_2	21.7 [7.1 - 107.1]	<0.0001
H7N9 vs. PW	α_2	37.3 [9.8 - 333.8]	<0.0001
H7N9 vs. CC/PW	ω	14.1 [5.5 - 45.4]	<0.0001
H7N9 vs. CC	ω	12.6 [4.0 - 62.9]	<0.0001
H7N9 vs. PW	ω	12.9 [4.1 - 64.5]	<0.0001

* amount of IFN neutralised, in serum diluted 1:50: 0.5 ng/ml IFNα_2, 0.2 ng/ml IFNω, 0.25 ng/ml IFNβ_{1b}
±, and/or; CC, close contacts; PW, poultry workers; CC/PW, healthy control groups combined; OR, odds ratio; CI, confidence interval; n.d., not determined

Table S8: Risk of H7N9 infection for individuals positive for IFN-I-neutralising autoantibodies relative to healthy control groups with adjustments for sex and age (high IFN concentrations)

Comparison	Autoantibodies neutralising*	OR [95% CI]	p value
H7N9 vs. CC/PW	$\alpha_2 \pm \omega \pm \beta_{1b}$	14.3 [6.0 - 34.2]	<0.0001
H7N9 vs. CC	$\alpha_2 \pm \omega \pm \beta_{1b}$	9.7 [3.5 - 26.8]	<0.0001
H7N9 vs. PW	$\alpha_2 \pm \omega \pm \beta_{1b}$	19.4 [5.3 - 71.3]	<0.0001

* amount of IFN neutralised, in serum diluted 1:50: 10 ng/ml IFNα_2, 10 ng/ml IFNω, 1 ng/ml IFNβ_{1b}
±, and/or; CC, close contacts; PW, poultry workers; CC/PW, healthy control groups combined; OR, odds ratio; CI, confidence interval

Table S9: Multivariable logistic regression model to investigate factors associated with fatality among H7N9 cases

Predictors	OR [95% CI]	p value
(Intercept)	0.42 [0.15 - 1.15]	0.096
Age	1.01 [0.99 - 1.03]	0.184
Male [Male]	0.84 [0.44 - 1.62]	0.602
neutralising_num	1.16 [0.55 - 2.43]	0.699
Observations	197	
R^2 Tjur	0.013	

OR, odds ratio; CI, confidence interval
